# 

**Published:** 1874-12

**Authors:** 


### To our Subscribers.

At the close of another year we send our greeting to each one of our subscribers, and wish them all a "Happy New Year." From the beginning of 1875 the new postal law requires us to pay postage on all our Journals, this will increase our expenses materially but we do not intend increasing our subscription rate, we will simply ask as a favor, that our friends will be prompt in responding to certain memoranda of their indebtedness to us which they have, or will in a short time receive.

Owing to uncontrollable circumstances our Journal has recently been late in its appearance ; we promise better for the future, and beg indulgence for the past. Please direct all communications and make all remittances payable to the Assistant Editor, Dr. E. N. Brush, No. 8, South Divisi n St., Buffalo, N. Y.

### Erie County Medical Society.

The Annual Meeting of this Society will be held January 12th, 1875, at the Medical College Hall. All members of the Society who have received Students into their offices without the certificates of the "Primary Board," will probably be expelled. It is hoped there will be a full attendance of all parties interested. The Senior Editor of this Journal, has received legal notice and specification of charges, and up to the present time, so far as we know he is the only member against whom charges have been preferred; others may therefore feel no alarm at the prospect of summary expulsion.

### Books and Pamphlets Received.

The Diseases of the Stomach. Being the third edition of the "Diagnosis and Treatment of the Varieties of Dyspepsia," Revised and Enlarged. By Wilson Fox, M. D., F.R.C.P., F.R.S. Philadelphia. Henry C. Lea, 1875. Buffalo: T. Butler & Son.

Outlines of the Science and Practice of Medicine. By Wm. Aitken, M. D., F.R.S. London: Charles Griffin & Co. Philadelphia: J. B. Lippincott & Co., 1874. Buffalo: T. Butler & Son.

Archives of Electrology and Neurology. A Journal of Electro-Therapeutics and Nervous Diseases. Edited by Geo. M. Beard, M. D. Vol. 1, No. 2.

Clinical Ureametry by Henry G. Piffard, M. D., from New York Medical and Surgical Journal, December 14.

Report of the Health Officer of the City and County of San Francisco, for the year ending June, 1874.

### Medical and Surgical Journal

PUBLISHED MONTHLY,

Containing Original Articles, Reports of Medical Societies and Hospitals. Editorial, Reviews, Correspondence, Army News, etc., etc; including the usual variety of Medical Periodical Publications. Specimen copies sent on application

Address Buffalo Medical & Surgical Journal, Buffalo, N. Y.

TERMS OF SUBSCRIPTION, - - $3.00 PER YEAR, IN ADVANCE.

